# Estimation of incubation period and serial interval of COVID-19: analysis of 178 cases and 131 transmission chains in Hubei province, China

**DOI:** 10.1017/S0950268820001338

**Published:** 2020-06-19

**Authors:** Lin Yang, Jingyi Dai, Jun Zhao, Yunfu Wang, Pingji Deng, Jing Wang

**Affiliations:** 1 School of Public Health and Management, Hubei University of Medicine, Shiyan, Hubei province, China; 2 Department of Infectious Diseases, The Third People's Hospital of Kunming City, Kunming, Yunnan province, China

**Keywords:** Coronavirus, epidemiology, incubation period, infectious disease, serial interval

## Abstract

A novel coronavirus disease, designated as COVID-19, has become a pandemic worldwide. This study aims to estimate the incubation period and serial interval of COVID-19. We collected contact tracing data in a municipality in Hubei province during a full outbreak period. The date of infection and infector–infectee pairs were inferred from the history of travel in Wuhan or exposed to confirmed cases. The incubation periods and serial intervals were estimated using parametric accelerated failure time models, accounting for interval censoring of the exposures. Our estimated median incubation period of COVID-19 is 5.4 days (bootstrapped 95% confidence interval (CI) 4.8–6.0), and the 2.5th and 97.5th percentiles are 1 and 15 days, respectively; while the estimated serial interval of COVID-19 falls within the range of −4 to 13 days with 95% confidence and has a median of 4.6 days (95% CI 3.7–5.5). Ninety-five per cent of symptomatic cases showed symptoms by 13.7 days (95% CI 12.5–14.9). The incubation periods and serial intervals were not significantly different between male and female, and among age groups. Our results suggest a considerable proportion of secondary transmission occurred prior to symptom onset. And the current practice of 14-day quarantine period in many regions is reasonable.

## Introduction

A novel coronavirus disease, designated as COVID-19, has become a pandemic worldwide. As of 29 March 2020, there were 634 835 confirmed COVID-19 cases and 29 957 deaths reported worldwide [1]. However, we still have a limited understanding about various aspects of our common enemy, such as the transmission dynamics [2]. Incubation period and serial interval are two key indicators that aid to depict the transmission dynamics of infectious infections [3]. Incubation period is the basis of the quarantine period for suspicious cases [4, 5]; while serial interval can help to estimate generation interval and reproduction number of an infectious disease [6, 7].

The reported median incubation periods of COVID-19 varied from 4 [8] to 8 days [9]. The mean serial interval estimated during the early outbreak of COVID-19 in Wuhan was 5.2 days [10], and 3.96 days in Mainland China outside Hubei province [11]. The above-mentioned studies have been limited by the small sample sizes and/or the fact that using publicly reported data. As a result, sampling bias and selection bias may have been introduced into the estimates [12].

To obtain reliable estimates of the incubation period and serial interval of COVID-19, we collected contact tracing data in a municipality in Hubei province during a full outbreak period. Our aim was to further assess the incubation period and serial interval of COVID-19, and to explore whether there were differences in incubation period and serial interval between cases with different characteristics.

## Methods

### Data

On 20 January 2020, the Shiyan Center for Disease Control and Prevention in Hubei province identified the first COVID-19 case and began monitoring people returning from Wuhan and its surrounding areas in Hubei province. The surveillance programme expanded to people who had recently returned from Wuhan regardless of symptoms; patients in hospitals or clinics; and individuals detected by fever screening in communities. Epidemiological surveys were conducted for each confirmed case using a standard format.

We collected data on cases with confirmed COVID-19 in Shiyan city based on the epidemiological contact tracing reports. Information collected included each case's sex, age, source of infection (imported from Wuhan, close contact with a Wuhan-imported case or locally infected), date of exposure (entry in and exit from Wuhan, or the earliest and latest date of close contact with a Wuhan-imported/locally infected case), date of symptom onset and date of diagnosis.

To estimate the incubation period and serial interval of COVID-19, the date of infection and transmission chain were inferred from the history of travel in Wuhan and/or history of exposure to confirmed cases, as follows: (i) if a patient had a history of travel in Wuhan within 2 weeks before symptom onset and stayed no more than 1 week in Wuhan, the left and right endpoints of the windows of possible date of infection were set as the date of entry into and exit from Wuhan, respectively; (ii) if a patient did not have a history of travel in Wuhan within 2 weeks before symptom onset and had a definite contact period with a confirmed case, the left and right endpoints of the windows of possible date of infection were set as the initial contact date and the last contact date, respectively; (iii) if a patient had a history of staying in Wuhan for more than 1 week, or if there were no clear contact information about a confirmed case, or if the contact period with a confirmed case was not clear, the date of infection was not recorded and (iv) if an infectee had no clear source infector or multiple possible source infectors, the transmission chain was not recorded.

### Statistical analysis

For each case, the date of infection was between two possible dates, while the date of symptom onset was definite. Therefore, for incubation period, such data are called single interval-censored data; for serial interval, they are exact observations. We calculated the incubation periods by imputing the infection date as the midpoint of each exposure interval, and explored the distribution of incubation period using a histogram and density plot. We then used an exponential probability distribution over the exposure interval for the time point of infection for each case implemented in the coarseDataTools [13] package. We fitted three commonly used distributions (Weibull, gamma and log-normal) [14–16] for the incubation period, and selected the best-fit model by comparing the log-likelihood values of these three types of models. The model with the largest log-likelihood value was selected as the best fit model. We calculated serial intervals as the time difference between dates of symptom onset of successive cases in a chain of transmission, and explored the distribution of serial interval using a histogram and density plot. We then fitted a normal distribution to the serial interval. The parameters and specific quantiles (5th, 50th, 95th and 99th percentiles) along with their bootstrapped 95% confidence intervals (CIs) were estimated for the fitted models.

To compare the incubation periods and serial intervals in cases with different characteristics, Wilcoxon rank sum tests and Kruskal–Wallis tests were used for the univariate analysis. We then split the data by type of case – Wuhan-imported cases *vs.* non-Wuhan-imported cases, and fitted two accelerated failure time models for the incubation period using sex and age as predictors.

Frequency counts and percentages were used in the descriptive analysis. *P* values <0.05 were considered significant in the inferential statistical analysis. All the analyses were performed using R software, version 3.6.1 [17].

## Results

### Patients' characteristics

Between 20 January 2020 and 29 February 2020, 672 COVID-19 cases were detected and diagnosed in Shiyan city, Hubei province (Table 1). Overall, there were approximately equal numbers of female and male cases (350 *vs.* 322), and more than half were adults between ages of 35 and 64. Among the 672 COVID-19 cases, 34 (5.1%) had no symptoms, 250 (37.2%) had a history of travel in Wuhan within 2 weeks before onset or diagnosis, 181 (26.9%) were close contacts of Wuhan-imported cases and 241 (35.9%) were locally infected. In the 41-day outbreak, most cases were diagnosed in late-January and the first week of February. By 22 March 2020, final clinical outcomes of all cases were known; with eight having died and 664 having recovered.

**Table 1.** Characteristics of COVID-19 cases in Shiyan city, Hubei province, China (*N* = 672)

Characteristics	*n* (%)
Sex	
Female	322 (47.9)
Male	350 (52.1)
Age (years)	
<14	38 (5.7)
14–34	189 (28.1)
35–64	355 (52.8)
>64	90 (13.4)
Symptom	
Yes	638 (94.9)
No	34 (5.1)
Source of infection	
Wuhan-imported	250 (37.2)
Close contact of imported cases	181 (26.9)
Locally infected	241 (35.9)
Time of diagnosis	
20 January to 31 January	216 (32.1)
1 February to 7 February	238 (35.4)
8 February to 29 February	218 (32.4)

### Estimation of incubation period and serial interval

**Fig. 1.**
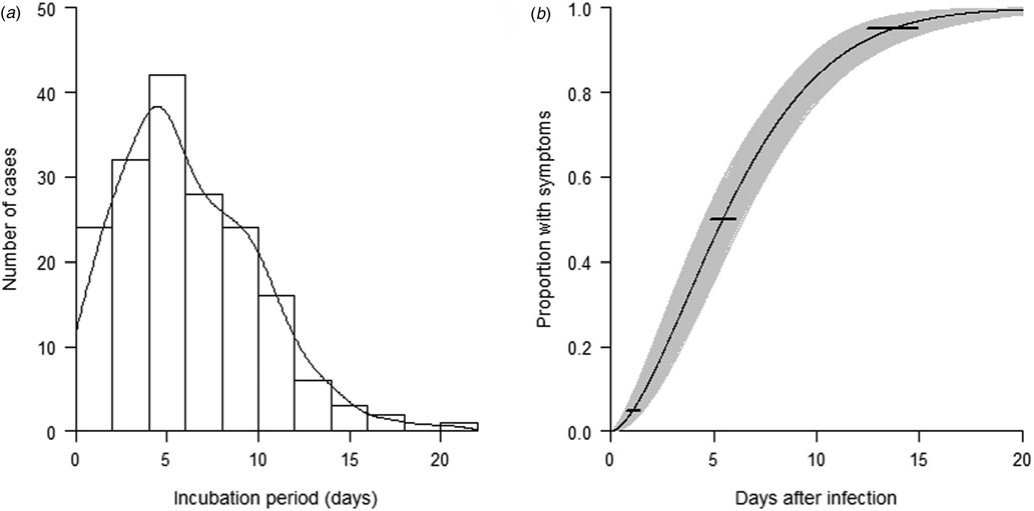
(a) Distribution of the observed incubation period of 178 COVID-19 cases. (b) Cumulative distribution function of the incubation period of COVID-19 under the Weibull distribution model. Horizontal bars represent the 95% CIs of the 2.5th, 50th and 97.5th percentiles of the incubation period distribution.

To estimate the incubation period of COVID-19, 178 cases with a clear-defined period of exposure and date of symptom onset were identified. Detailed exposure to symptom onset timeline for these 178 cases is presented in Supplementary Fig. 1. The length of the intervals used to determine exposure window varied from 1 to 7 days. There was no significantly difference in different sources of infection (*P* value = 0.47). The observed incubation periods showed a right skewed distribution with a median of 6 days (range: 1–21) (Fig. 1a). After comparing the log-likelihood values of the Weibull distribution, gamma distribution and log-normal distribution, we found that Weibull model best fit the observed incubation periods (Fig. 1b). From the fitted model, the estimated incubation period of COVID-19 falls within the range of 1–15 days with 95% confidence, and it has a median of 5.4 days with a bootstrap 95% CI 4.8–6.0 days. Of the symptomatic cases, 5% showed symptoms by 1.1 days (95% CI 0.8–1.4) after infection, 95% showed symptoms by 13.7 days (95% CI 12.5–14.9) and 99% showed symptoms by 17.8 days (95% CI 15.9–19.7) (Table S1).

**Fig. 2.**
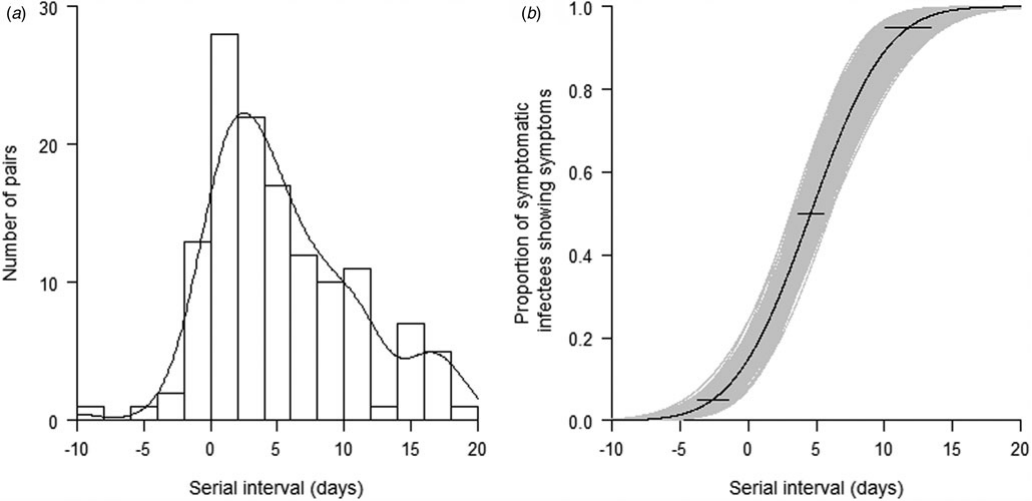
(a) Distribution of the observed serial interval of 131 pairs with confirmed close contact. (b) Cumulative distribution function of the serial interval of COVID-19 under the Weibull distribution model. Horizontal bars represent the 95% CIs of the 2.5th, 50th and 97.5th percentiles of the serial interval distribution.

To estimate the serial interval of COVID-19, 152 pairs of cases with a clear infector–infectee relationship were identified. These 152 pairs included 233 individuals. All infectees (secondary cases) were uniquely linked to an infector (source case), but 12 infectors were linked to two infectees, 10 infectors to three infectees and two infectors to more than three infectees. Of these 233 cases, 202 (131 pairs) had complete information on the date of symptom onset. The transmission timeline for these 131 infector–infectee pairs is presented in Supplementary Fig. 2. We calculated the serial interval for each of these 131 pairs. We observed 10 (7.6%) negative serial intervals which indicates that the infectees showed symptoms prior to their infectors. The serial intervals showed an approximately normal distribution with a median of 4 (range: −10 to 19) days (Fig. 2a). We then fitted a normal distribution to the observed serial intervals (Fig. 2b). From the fitted model, the estimated serial interval of COVID-19 has a mean of 4.6 days (95% CI 3.7–5.5) and a standard deviation of 4.4 days (95% CI 3.8–5.0). Of the symptomatic cases, 95% of infectees showed symptoms by 11.8 days (95% CI 9.7–13.9), and 99% showed symptoms by 14.8 days (95% CI 12.1–17.5) (Table S1).

### Stratified analysis of incubation period and serial interval

A comparison of incubation periods and serial intervals in cases with different characteristics is presented in Table 2. No significant differences in incubation period between male and female and among different age groups were observed. However, the incubation period was significantly associated with the source of infection. There was a general increasing trend of incubation period from the source cases to the second (close contact of Wuhan-imported cases) and third (locally infected) generation cases. The infectees' sex, age and source of infection were not found to be significantly associated with serial intervals. In the multivariate Weibull regression model stratified by Wuhan-imported cases and non-Wuhan-imported cases, the incubation periods were not found significant different between male and female, and among different age groups (Table 3).

**Table 2.** Univariate association between characteristics of COVID-19 cases and incubation periods and serial intervals

	Incubation period			Serial interval		
	Cases (*n* = 178)	Median (IQR)	*P* value*	Secondary cases (*n* = 131)	Median (IQR)	*P* value*
Sex			0.848			0.900
Female	89	6 (4, 9)		73	5 (2, 8)	
Male	89	6 (4, 9)		58	4 (1.25, 9)	
Age (years)			0.054			0.358
<14	3	8 (7, 8.5)		10	1 (1, 5.25)	
14–34	43	5 (3, 7.5)		24	5 (2, 6)	
35–64	17	6 (4, 9)		75	5 (2, 9)	
>64	25	9 (6, 11)		22	4 (2, 9.5)	
Source of infection			0.009			0.952
Wuhan-imported	42	5 (2, 7.75)		8	4.5 (2.75, 7.5)	
Contact Wuhan cases	99	6 (4, 9)		68	4 (1, 11)	
Locally infected	37	7 (5,10)		55	4 (2, 8.5)	

*P values were obtained from Wilcoxon rank sum test for sex, and Kruskal–Wallis test for age and source of infection.

**Table 3.** Association between incubation periods and characteristics of COVID-19 cases by source of infection in multivariate Weibull regression models

	Wuhan-imported		Non-Wuhan-imported	
	Time ratio (95% CI)	*P* value	Time ratio (95% CI)	*P* value
Sex				
Female	Reference		Reference	
Male	1.11 (0.73–1.68)	0.637	1.00 (0.84–1.19)	0.998
Age (years)				
<35	1.00 (0.65–1.53)	0.994	1.10 (0.881.37)	0.420
35–64	Reference		Reference	
>64	1.37 (0.60–3.11)	0.452	1.24 (0.98–1.58)	0.079

CI, confidence interval.

*P values were obtained from Wald tests.

## Discussion

Our analysis of the incubation period and serial interval of COVID-19 used contact tracing data and accounted for interval censoring of exposures. Our study shows that the incubation period of COVID-19 follows a Weibull distribution, and the serial interval follows an approximately normal distribution. The median incubation period was 5.4 days, and the median serial interval was 4.6 days.

Our estimate of the median incubation period (5.4 days) is longer than the values from two studies conducted in the early outbreak of COVID-19, 5.2 [10] and 4.8 days [18], respectively. These two studies set the date of leaving Wuhan as the exposure time. However, because of the exponentially increasing incidence in Wuhan throughout December and January, infection was much more likely to have occurred towards the end of exposure intervals, which would lead to underestimation of the incubation period. On the other hand, our estimate of the median incubation period (6.0 days) is shorter than the value of 6.4 days based on publicly reported data [11, 12].

Time of infection is crucial in determining the incubation period. The Dongfeng Motor Corporation, founded in Shiyan in 1969, is now headquartered in Wuhan [19]. However, many of its manufacturing plants are still in Shiyan. As a result, there is more frequent population mobility between the two cities than other cities in the province. This facilitated our ability to estimate the time of infection in cases with a history of short stay in Wuhan before symptom onset. Besides, after Wuhan announced it was shutting down on 23 January 2020, Shiyan shut down its traffic 2 days later, and then closed communities and villages the following day, on 26 January 2020. As a result, most transmissions of COVID-19 were developed within families or neighbourhoods. Therefore, for those close contacts of Wuhan-imported cases, we could infer the transmission chain and the time of infection with high accuracy. For these reasons, our estimate of the incubation period and serial interval should be more justifiable than those in previously published studies.

The estimated median incubation period for COVID-19 of 6.0 days is longer than those for other coronaviruses that have been discovered so far: for SARS, 4.0 days (95% CI 3.6–4.4) [20], and for MERS, 5.0 days (95% CI 3.6–4.4) [21]. According to our results, 95% of symptomatic cases will show symptoms within 13.7 days. This supports the currently practiced 2-week quarantine in many countries.

The estimated median serial interval in this study (4.6 days, 95% CI 3.7–5.5) is very close to the value of 3.96 (95% CI 3.53–4.39) based on 468 infector–infectee pairs [11] and 4.6 (95% CI 3.5–5.9) based on 28 infector–infectee pairs [22]. The median serial interval of COVID-19 is shorter than the disease's median incubation period suggesting that a considerable proportion of transmissions occurs before symptom onset. This is consistent with the result from a previous study [22]. The pre-symptomatic transmissions suggest that a containment via case isolation alone is likely to be very challenging. Shiyan city was locked down on 26 January, families were confined to their homes. Therefore, most transmissions occurred within families, leading to more frequent and faster transmission within homes than in the community. The city started the surveillance programme in the first week of the outbreak. Exposed individuals identified by contact tracing were isolated in the designated hotels. Thus, a larger fraction of transmissions would be occurring before symptom onset of the infectors. As a result, the contact tracing activities would reduce the length of pre-symptomatic transmissions, and lead us to observe relatively short serial intervals.

The present study has several limitations. First, the data were extracted from contact tracing reports. Recall bias was inevitable, particularly for the key timing variables. Second, we only had information on confirmed cases and may have missed some cases with very mild symptoms. In addition, we limited the exposure time of Wuhan-imported cases to within 2 weeks before symptom onset or travelled in Wuhan for more than 1 week to obtain more reliable exposure time periods. A large proportion of Wuhan residences were excluded. This leads to a bias against identifying longer incubation periods in the Wuhan-arrivals.

In conclusion, our results contribute to a better understanding of COVID-19 and provide useful parameters for modelling the dynamics of disease transmission. A considerable proportion of secondary transmission occurred prior to symptom onset. The current practice of a 14-day quarantine period in many regions is reasonable. Considering that there are still approximately 5% of cases with incubation periods of more than 14 days, it is necessary to maintain social distance and wear a mask for a certain period after the quarantine.

## Data Availability

The data that support the findings of this study are openly available in ‘Zenodo’ at http://doi.org/10.5281/zenodo.3898225.

## Supplementary material

For supplementary material accompanying this paper visit http://dx.doi.org/10.1017/S09502688200013381. click here to view supplementary material
