# Supplementary figures and images for: Estimation of incubation period and serial interval of COVID-19: analysis of 178 cases and 131 transmission chains in Hubei province, China
Source: Epidemiol Infect. 2020 Jun 19;148:e117. doi: 10.1017/S0950268820001338 (PMC7324649; doi:10.1017/S0950268820001338)

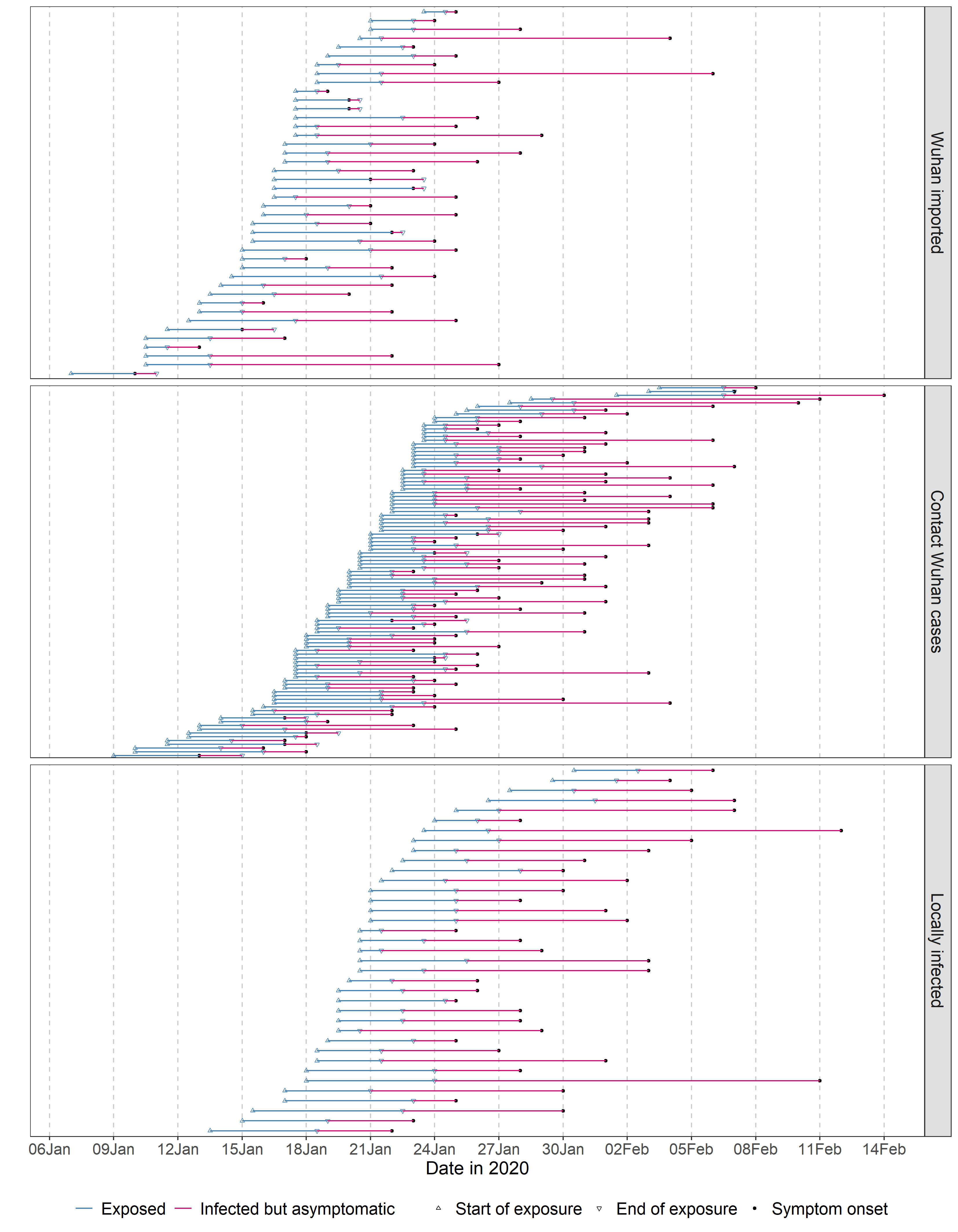

Supplement: Supplementary file 1 [file S0950268820001338sup.zip › S0950268820001338sup001.tiff.tif]

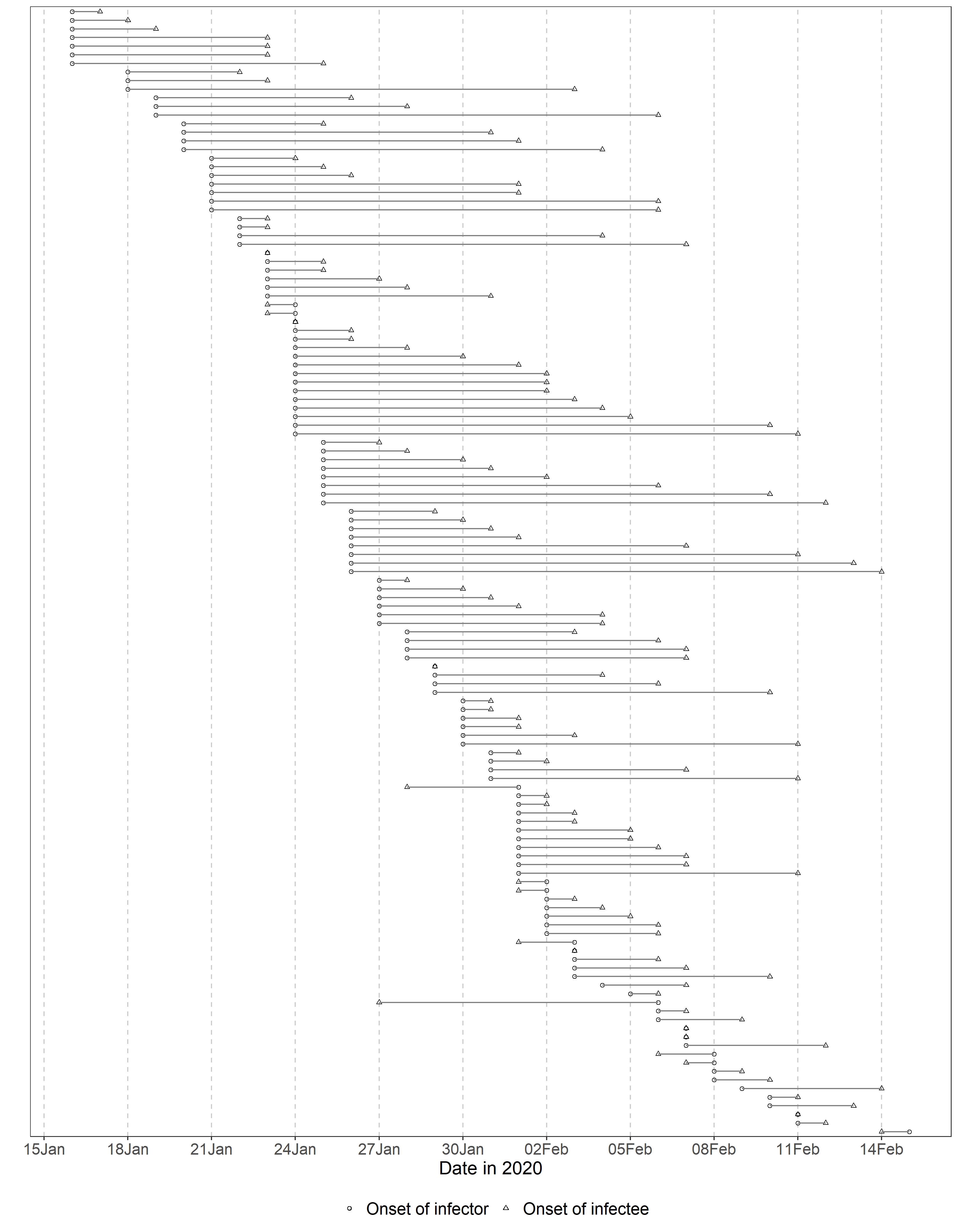

Supplement: Supplementary file 1 [file S0950268820001338sup.zip › S0950268820001338sup002.tiff.tif]
